# Transporters in plant sulfur metabolism

**DOI:** 10.3389/fpls.2014.00442

**Published:** 2014-09-09

**Authors:** Tamara Gigolashvili, Stanislav Kopriva

**Affiliations:** ^1^Department of Plant Molecular Physiology, Botanical Institute and Cluster of Excellence on Plant Sciences, Cologne Biocenter, University of CologneCologne Germany; ^2^Plant Biochemistry Department, Botanical Institute and Cluster of Excellence on Plant Sciences, Cologne Biocenter, University of CologneCologne Germany

**Keywords:** sulfate transporter, cysteine (Cys) transporter, methionine (Met) transporter, glutathione (GSH) transporter, S-adenosylmethionine (SAM) transporter, 3′-phosphoadenosine-5′-phosphosulfate (PAPS) transporter, 3′-phosphoadenosine 5′-phosphate (PAP) transporter, transporter of glucosinolates (GSL)

## Abstract

Sulfur is an essential nutrient, necessary for synthesis of many metabolites. The uptake of sulfate, primary and secondary assimilation, the biosynthesis, storage, and final utilization of sulfur (S) containing compounds requires a lot of movement between organs, cells, and organelles. Efficient transport systems of S-containing compounds across the internal barriers or the plasma membrane and organellar membranes are therefore required. Here, we review a current state of knowledge of the transport of a range of S-containing metabolites within and between the cells as well as of their long distance transport. An improved understanding of mechanisms and regulation of transport will facilitate successful engineering of the respective pathways, to improve the plant yield, biotic interaction and nutritional properties of crops.

## INTRODUCTION

Sulfur (S) is as an essential macronutrient for plant growth, development, and response to environmental changes. It is required for the biosynthesis of proteins, co-enzymes, prosthetic groups, vitamins, amino acids like Cys and Met, GSH, and secondary metabolites such as GSL and sulfoflavonoids. Understanding S metabolism in plants is essential for human nutrition as Met is an essential amino acid and as many S containing secondary metabolites and specialized peptides [i.e., GSH and PC] are important for biotic and abiotic interactions of crop plants and their yield. Sulfur uptake and distribution is tightly controlled in response to developmentally or environmentally induced changes in nutrient demand (Yoshimoto et al., 2003; Buchner et al., 2004a). Although the primary and secondary sulfur assimilation pathways are well described, the metabolic networks and fluxes of these metabolites are still poorly understood (Gläser et al., 2014).

Taken up from the soil, sulfate is incorporated into adenosine-5′-phosphosulfate followed by reduction into sulfite and then sulfide and Cys biosynthesis. In parallel, adenosine-5′-phosphosulfate can be further phosphorylated to 3′-phosphoadenosine-5′-phosphosulfate, which is used for sulfation reactions (Mugford et al., 2011; Ravilious and Jez, 2012). Cys is the key metabolite in the synthesis of sulfur-containing compounds in plants, while the major pool of sulfur, which is not stored in proteins is the Cys-containing peptide GSH (Hell and Wirtz, 2011). GSH is a universal molecule, which plays a crucial role in plants including cellular defense, redox status, signal transduction and detoxification (Noctor et al., 2012). GSH forms conjugates with electrophilic compounds such as heavy metal ions, secondary metabolites or xenobiotics via sulfhydryl group – the reaction mediated by GSH S-transferases or happening spontaneously (Edwards and Dixon, 2005; Cummins et al., 2011).

About 300 different metabolites are predicted to incorporate sulfur in *Arabidopsis* and about 140 more sulfur metabolites that have not been assigned to the databases to date have been reported recently (Gläser et al., 2014). These metabolites are derived from various metabolic pathways and have diverse functions that range from proteogenic amino acids (Cys, Met), hormone derivatives (e.g., sulfojasmonate and sulfated brassinosteroids), antioxidants (e.g., GSH), signaling molecules (phosphonucleotide, PAP, and H_2_S), and secondary metabolites (GSLs, sulfoflavonoids). Given the large number of metabolites within S-assimilation pathway, and the localization of enzymes and pathways in different compartments, a wide spectrum of plant metabolite transporters has to be expected. Plants have evolved a network of transporters to maintain homeostasis of sulfur and S-derived compounds. Specific intra-and inter-cellular transporters are needed to store the sulfur or to channel it in biochemical processes allowing biosynthesis of important S-containing metabolites.

Despite their importance for sulfur homeostasis, our knowledge of intracellular and intercellular transporters in S assimilation is still limited. In recent years, significant progress has been made in elucidating the functions of some carriers important in S-metabolism in plants. Still, many transport proteins remain unidentified. This review provides an overview of known transport proteins in sulfur metabolism within the cell and plant as a whole and aims understanding of their role in the maintenance of sulfur levels in plants.

## SULFATE TRANSPORTERS

Sulfate transporters are the most prominent group of S-metabolite transporters in plants because sulfate is the major source of sulfur taken up from the soil and because it is the most abundant S-containing metabolite in plant cells. Accordingly, the first cloned gene for a transporter of sulfur metabolite in plants was a gene for SULTR (Smith et al., 1995). In this pioneering work the authors used the complementation of yeast mutant unable to take up sulfate to isolate three different cDNA clones for SULTRs from *Stylosanthes hamate*, a tropical forage legume. These cDNAs encoded two of high affinity transporters and one a low affinity transporter (Smith et al., 1995).

Functionally, these proteins are H^+^/sulfate co-transporters, which corresponds with their phylogenetic relation with SULTRs from other organisms. SULTRs can be divided into three major groups according to their mechanisms, ATP-dependent ABC type transporters, Na^+^(H^+^)/sulfate symporters, and sulfate/anion (Cl^-^, CO_3_^2-^, oxalate) antiporters (Markovich and Aronson, 2007; Ohana et al., 2009; Takahashi et al., 2011a). The two latter groups are represented by the SLC13 and SLC26 gene families, respectively, (Takahashi et al., 2011a). In higher plants, only the H^+^/SULTRs are present, while in green algae and many microalgae genes all three groups are present (Takahashi et al., 2011a; Bochenek et al., 2013). ABC-type of SULTRs are known to import sulfate to plastids of green algae but are not present in Bryophytes and seed plants (Melis and Chen, 2005). Plant SULTRs are evolutionary related to the SLC26 group, but with a reaction mechanism of H^+^/sulfate symport, similar to the SLC13 group, which use Na^+^ (Takahashi et al., 2011a). They are integrated into the membranes by 12 transmembrane regions and contain a STAS domain, found in SULTRs and with a significant similarity to bacterial anti-sigma factor antagonists (Takahashi et al., 2011a,b). The STAS domain is important for the correct incorporation into the membrane, activity, and interaction with other proteins (Shibagaki and Grossman, 2004, 2006).

Already the first report of cloning of plant SULTRs demonstrated that they are encoded by multigene family (Smith et al., 1995). *Arabidopsis* possess 12 *SULTR* genes, whereas 11 genes are present in rice, 13 in poplar, and 5 *SULTR* genes are encoded in the sequenced genomes of basal plants *Selaginella moellendorffii* and *Physcomitrella patens* (Kopriva et al., 2009; Takahashi et al., 2011a). The transporters can be divided in four distinct groups, which are also functionally divergent. The first group encodes high affinity SULTRs, group 2 are low affinity transporters, group 4 encodes vacuolar sulfate exporters, and the group 3 is the most diffuse from these groups, encoding transporters of the plastid membranes, symbiosome membranes, and others with specific or unknown functions (Buchner et al., 2004b; Takahashi et al., 2011b). Every plant species possesses in addition one or two genes with a significant sequence similarity to SULTR, but lacking the STAS domain. These genes were traditionally included into the SULTR family as group 5, but since they were shown to be involved in transport of molybdate and could never be confirmed to transport sulfate (Tejada-Jimenez et al., 2007; Tomatsu et al., 2007; Baxter et al., 2008), they are not considered to be SULTRs any more (Takahashi et al., 2011a).

**FIGURE 1:**
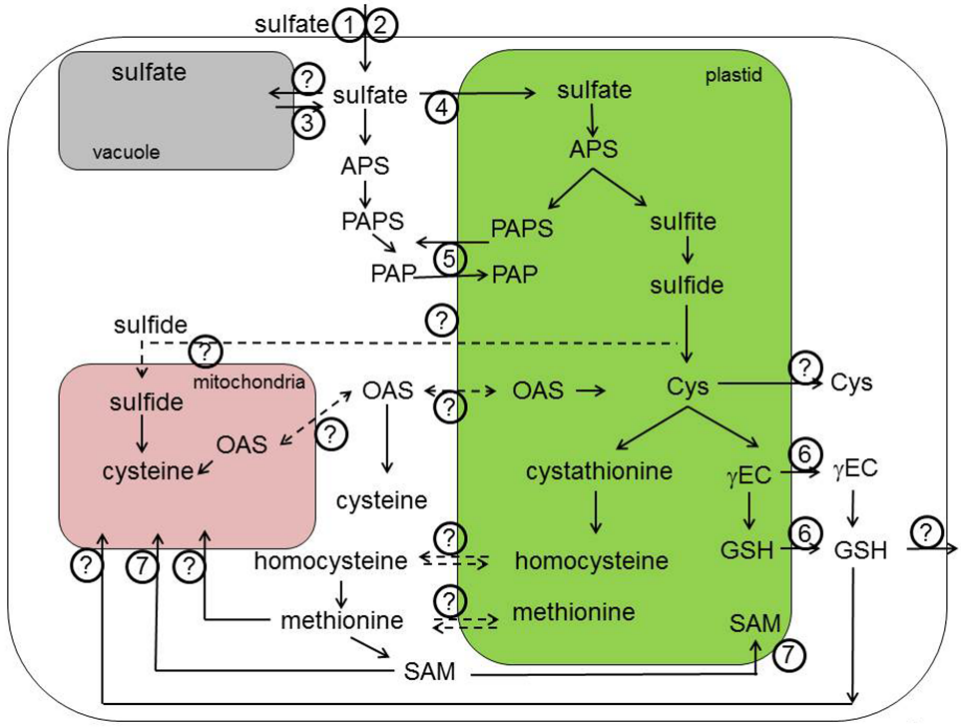
** Transport processes in primary sulfur assimilation.** Sulfate is taken up by the root cells with the help of SULTR1;1 **(1)** and SULTR1;2 **(2)**. Once crossed the plasma membrane of epidermal and cortical root cells, sulfate is transported through the series of sulfate transporters (SULTR) residing in various membranes within the plant. The SUTR4;1 and SULTR4;2 are important for the efflux from the vacuole into the cytoplasm **(3)**. The transporter important for the sulfate influx into vacuole is still unknown. Import of sulfate into the chloroplasts is possible due to SULTR3;1 and probably other members of SULTR3 subfamily **(4)**. PAPS is produced both in chloroplasts and the cytoplasm and can be exchanged between these compartments by PAPST/TAAC transporter **(5)**. The known transporter of thiols (GSH and γEC) are chloroquine-resistance transporter (CRT)-like proteins or CRLs **(6)**. However, an alternative transport systems for thiols in the plastid membrane is expected to exist. In a similar way, the GSH transporters to the mitochondria await still the discovery. S-adenosylmethionine transporter 1 (SAMT1; **7**) is a chloroplastidic protein involved in the exchange of SAM with S-adenosylhomocysteine, the by-product of methylation reactions that has to be regenerated to SAM in the cytoplasm. This is also the case for the plasmalemma localized transporters of *S*-methylmethionine (SMM) and GSH, which are important transport form of reduced sulfur and therefore need to be exported out of the cell. APS, adenosine 5′-phosphosulfate; Cys, cysteine; OAS, *O*-acetylserine; γEC, γ-glutamylcysteine; GSH, glutathione; SAM, S-adenosylmethionine; PAPS, 3′-phosphoadenosine-5′-phosphosulfate; PAP, 3′-phosphoadenosine 5′-phosphate. Dashed lines indicate theoretically possible transport pathways.

The SULTR family is best characterized in *Arabidopsis*. Three genes form the group 1, *SULTR1;1,* and *SULTR1;2* are expressed in roots and are responsible for sulfate uptake from the soil (**Figures 1 and 3**, transporters 1–4 and 13–15, respectively). Plants lacking both these transporters are unable to take up sulfate in low concentrations and are strongly affected in growth (Yoshimoto et al., 2002, 2007; Rouached et al., 2008). The transporters have overlapping function, but are differentially regulated, with the SULTR1;1 playing an important role during sulfate starvation (Rouached et al., 2008). On the other hand, during normal sulfate supply, SULTR1;2 is the more prominent transporter, as evidenced from the experiments showing selenate resistance of *sultr1;2* mutants (Shibagaki et al., 2002). In addition, SULTR1;2 has been proposed to act as sensor of sulfur status of plants (Zhang et al., 2014), but more evidence is necessary to dissect the mechanism of such sensing. SULTR1;3 is a high affinity transporter localized in phloem and important for source–sink redistribution of sulfate (Yoshimoto et al., 2003). The two low affinity group 2 transporters are localized in vasculature and are responsible for long distance translocation of sulfate (Takahashi et al., 2000). Group 4 transporters are found in tonoplast and facilitate sulfate efflux from the vacuoles (Kataoka et al., 2004b). The first group 3 transporter characterized was SULTR3;5, which was shown to modulate the function of SULTR2;1 but not to transport sulfate itself (Kataoka et al., 2004a). The other members of this group were shown recently to be present in the plastid envelope and to catalyze sulfate import to the plastids (Cao et al., 2013). Interestingly, all *SULTR* transcripts with exception of root specific *SULTR1;1* were highly and coordinately enriched in bundle sheath cells of *Arabidopsis* leaves (Aubry et al., 2014). **Figure 1** shows the known (transporters 1–4) and yet to be identified SULTRs localized in various membranes within the plant cell.

Other higher plant species have similar structure of the SULTR family, whereas in lower plants only the group 4 is distinct (Kopriva et al., 2009). The structures and transcriptional regulation of whole SULTR families were characterized from several plant species, *Brassica oleracea*, poplar, wheat, and *Medicago truncatula* (Buchner et al., 2004a, 2010; Durr et al., 2010; Casieri et al., 2012). In addition, the transporters from *S. hamate* also follow the pattern of the functional groups, the two high affinity transporters are similar to other group 1 transporters and the low affinity transporter belongs to group 2. High affinity SULTR characterized in barley belongs to group 1 (Smith et al., 1997). In accordance with the “diffuse” functions of group 3, a SST1 transporter in symbiosome membranes of *Lotus japonicus*, which is essential for proper function of nodules, belongs to group 3 (Krusell et al., 2005) and a SULTR3;5 from poplar seems to be involved in interaction with fungal pathogens (Petre et al., 2012).

## REGULATION OF SULFATE TRANSPORT

Sulfate uptake plays a major role in the control of plant sulfur homeostasis (Vauclare et al., 2002). This is evident from the response of sulfate uptake to sulfur availability and its regulation by environmental conditions. Sulfate uptake is induced under sulfate limiting conditions and is repressed in the presence of reduced sulfur (Smith et al., 1995, 1997; Takahashi et al., 1997, 2011b; Yoshimoto et al., 2002). Sulfate uptake is coordinated with the uptake of nitrate, so at low nitrate levels and with availability of carbohydrates sulfate uptake is repressed (Smith, 1980; Brunold, 1993; Koprivova et al., 2000; Kopriva et al., 2002). Particular significance for overall control of plant sulfur nutrition has regulation by the precursor of Cys, (Smith et al., 1997; Hopkins et al., 2005). Many experiments showed that the regulation of sulfate uptake is well correlated with regulation of mRNA levels of the transporters, in particular those of group 1 (Smith et al., 1997; Takahashi et al., 1997, 2011b; Abola et al., 1999; Yoshimoto et al., 2002; Rouached et al., 2008). Indeed, sulfate starvation, which results in increased sulfate uptake, induces transcript levels of *Arabidopsis SULTR1;1*, *1;2*, *2;1*, *4;1*, and *4;2* (Takahashi et al., 1997, 2000; Abola et al., 1999; Yoshimoto et al., 2002; Kataoka et al., 2004b). The same is true for other plant species, increase of *SULTR* transcript levels was observed in sulfur starved *Brassica*, wheat, *Medicago*, barley, etc. (Smith et al., 1997; Abola et al., 1999; Buchner et al., 2004a, 2010; Koralewska et al., 2009; Casieri et al., 2012). OAS, which accumulates in sulfur starved plants, induces mRNA levels of *SULTR* genes even at sufficient sulfate supply (Smith et al., 1997; Hirai et al., 2003; Hopkins et al., 2005), and may so act as a signal in the sulfate starvation regulatory network (Hirai et al., 2005; Hubberten et al., 2012). The transcript levels of SULTR are rapidly reduced when sulfate is resupplied to sulfur starved plants (Koralewska et al., 2009).

Sulfate starvation is one of the best analyzed environmental condition using systems biology approaches (Hirai et al., 2003; Maruyama-Nakashita et al., 2003; Nikiforova et al., 2003, 2004; Hubberten et al., 2012). The efforts to dissect the mechanisms of the regulation resulted in identification of at least some components of the regulatory circuits. One *cis* and one *trans* factor important for the regulation of *SULTR* genes have been identified. Analysis of promoter of *SULTR1;1* gene revealed a presence of a 16-bp SURE, present in promoters of many S-starvation inducible genes (Maruyama-Nakashita et al., 2005). The transcription factor SLIM1 has been identified by a genetic screen using *SULTR1;2::GFP* as reporter construct (Maruyama-Nakashita et al., 2006). SLIM1 is a member of the EIL family transcription factors, ETHYLENE-INSENSITIVE3-LIKE3 (EIL3), and controls the sulfate starvation response of a large number of, but not all, responsive genes (Maruyama-Nakashita et al., 2006; Kawashima et al., 2011). Since *SLIM1* mRNA is not affected by S-starvation, the mechanisms of its action is not known, it is also not clear whether it actually binds to the SURE element. However, experiments with phosphatase inhibitors revealed that dephosphorylation is a part of the signal transduction (Maruyama-Nakashita et al., 2004a) and that cytokinins may be important for controlling *SULTR1;1* expression (Maruyama-Nakashita et al., 2004b).

The regulation of sulfate uptake is, however, more complex and includes also post-transcriptional mechanisms. In *sultr1;1 sultr1;2* plants complemented by constitutively expressed *SULTR1;1* and *1;2*, accumulation of the transporters was induced by S-starvation despite the mRNA accumulation being not affected (Yoshimoto et al., 2007). Thus, the induction of sulfate uptake is not completely derived from upregulation of the transcript but another, unknown, post-transcriptional mechanism is necessary for proper regulation. Another post-transcriptional mechanism to improve sulfate uptake during S-starvation affects different SULTR isoform, the low affinity SULTR2;1. The SULTR2;1 is a target of microRNA miR395, which is induced by S-starvation in a SLIM1-dependent manner (Kawashima et al., 2009, 2011). The function of SULTR2;1 in response to S-starvation is to increase the translocation of sulfate from the roots to the shoots. The mechanism of the miR395 regulation of SULTR2;1 is, however, non-canonical, as both the miRNA and its target are actually induced by S-starvation. The spatial expression patterns of the two transcripts are non-overlapping, so that miR395 restricts SULTR2,1 expression to the xylem parenchyma cells and prevents its accumulation in phloem companion cells. This expression pattern of SULTR2;1 increases the efficiency of xylem loading, to increase translocation of sulfate to the leaves and prevents the unloading from phloem back to the roots (Kawashima et al., 2009). The third post-translational mechanism of regulation of sulfate uptake is the modulation of SULTR1;2 activity through interaction with the Cys synthase in cytoplasm. The Cys synthase binds to the STAS domain and reduces the activity of the transporter; at the same time the interaction results in increased activity of the Cys synthase (Shibagaki and Grossman, 2010).

Despite the impressive progress in understanding of plant sulfate transport, and the recent identification of long-sought plastidic SULTR (Cao et al., 2013), many questions remain open. The transporters responsible for sulfate influx into vacuoles are still not known. The redundancy of the plastidic transporters SULTR3;1-3;4 is in contrast to the specific functions of the other *SULTR* genes and a detailed dissection of their individual function still has to be undertaken. Given the subtle effects of *sultr3* mutants on seeds (Zuber et al., 2010), this might not be a trivial task. The possible function of SULTR1;2 (Zhang et al., 2014) as a sulfate sensor is intriguing, particularly as the *Chlamydomonas* regulator of S-starvation response, SAC1, is similar to SULTR of the SLC13 group (Davies et al., 1996; Takahashi et al., 2011a). The molecular mechanisms of regulation of SULTR need to be elucidated; apart from SLIM1 and HY5, no other transcription factors binding the *SULTR1;2* promoters have been reported (Maruyama-Nakashita et al., 2006; Lee et al., 2011). Sulfate transport will thus further remain in prime focus of investigations of plant sulfur metabolism.

## TRANSPORT OF CYSTEINE AND GLUTATHIONE

**FIGURE 2:**
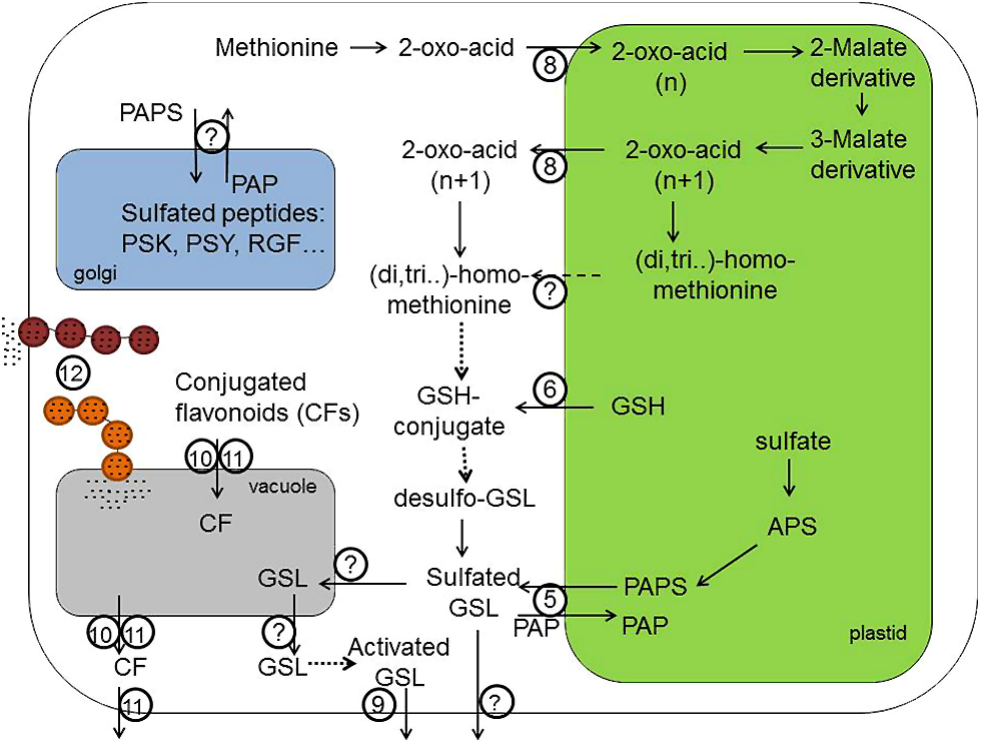
**Transport processes in the biosynthesis of sulfur containing GSLs, CF and sulfated peptides like PSK, PSY, and RGF.** 3′-phosphoadenosine-5′-phosphosulfate (PAPS) required for the sulfation of secondary GLS and sulfated flavonoids is produced in plastids and is exported by (**5**; PAPST1/TAAC transporter) into the cytoplasm, where sulfotransferases can produce sulfated secondary metabolites. Transporter **(6)** probably provides GSH required for the biosynthesis of GSL core structure and secondary modification of phytochemicals localized in the cytoplasm. Transporter **(8)** – is a bile acid transporter 5 (BAT5) – involved in the exchange of 2-oxo-acids like 4-methylthio-2-oxobutanoate-MTOB, 5-methylthio-2-oxopentanoate – MTOP, 6-methylthio-2-oxohexanoate – MTOH between plastid and cytoplasm. However, not only the export of 2-oxo-acids but also the export of side-chain elongated methionine (homoMet, dihomoMet, trihomoMet, etc.) from chloroplasts is theoretically possible. The identity of this transporter remains, however, unknown. Transporter **(9)** is a PEN3/PDR8 belonging to pleiotropic drug resistance (PDR) subfamily of ABC transporter family, which is expected to transport the highly potent catabolism products of indolic GSL across plasma membrane into apoplastidic space and at the side of pathogen entry. Transporters **(10)** and **(11)** are importers of sulfated and glutathionilated flavonoids transporting these conjugated flavonoids (CFs) across tonoplast. There are two different classes of transporters – the multidrug resistance-associated protein (MRP) belonging to ABC transporter family **(10)** and the multidrug and toxic compound extrusion transporters (MATE; **11**). The transporter **(11)** can additionally export conjugated flavonoids from the vacuole and across plasma membrane into apoplastidic space. **(12)** Transport Mediated by Vesicle Trafficking. The importer of GSL into vacuole should exist but could not be identified so far. Also plasmalemma-localized exporters of native GSL from the cell are not known. For the tyrosine sulfation of hormone-like peptides (PSKs, PSY, and RGFs) in Golgi apparatus, the PAPS need to be imported into the Golgi lumen. However, an identity of these PAPS transporters has not been discovered yet. APS, adenosine 5′-phosphosulfate; GSH, glutathione; PAPS, 3′-phosphoadeno sine-5′-phosphosulfate; PAP, 3′-phosphoadenosine 5′-phosphate; GSL, gluocosinolates; PSK, phytosulfokine; PSY, plant peptide containing sulfated tyrosine; RGF, root growth factor. Dashed lines indicate theoretically possible transport pathways. Dotted lines indicate biochemical reactions requiring several steps for the conversion of substrates.

Intracellular transport of amino acids is essential, as in plants protein synthesis occurs in three organelles. In has been hypothesized, however, that this may not to be the case for Cys, as Cys synthesis is also localized in these three compartments, cytosol, mitochondria, and plastids (Lunn et al., 1990). However, analysis of mutants in the OASTL revealed that the Cys synthesis can be restricted to a single compartment without affecting survival and with only small effects on growth (Heeg et al., 2008; Watanabe et al., 2008; Birke et al., 2012). Thus, Cys or its precursor must be transported across the mitochondrial and plastid membranes in both directions (**Figures 1 and 2**, the identity is unknown). Investigations of Cys transport across mitochondrial membrane revealed presence of multiple transport systems with different kinetic properties (Lee et al., 2014), but specific transporters have not been reported in plants. Cys also undergoes intercellular transport, although its contribution to a total long-distance flow of sulfur may not be very high (Herschbach and Rennenberg, 1995). For example, seeds are able to assimilate sulfate and are therefore not dependent on transport of Cys (Tabe and Droux, 2001). In C4 plants, however, sulfur nutrition is dependent on intercellular Cys transport, since sulfate is reduced in the bundle sheath cells only and Cys is the transport metabolite from these cells to mesophyll and other cell types of the leaves (Burgener et al., 1998; Kopriva and Koprivova, 2005). Plants possess a large number of amino acid transporters, many of them capable of transporting Cys, some even with a high specificity (Miranda et al., 2001; Tegeder, 2012). In yeast, Cys can be transported by at least eight unspecific amino acid permeases, but the major contributor is a specific YCT1 (Kaur and Bachhawat, 2007). In animals multiple systems transport Cys rather than Cys (McBean and Flynn, 2001). It is not clear whether similar specific Cys (or Met) transporters exist in plants or whether Cys transport is less specific through general amino acid permeases. Thus, the molecular nature of Cys transport into the cells as well as in mitochondria and plastid membranes still remains to be elucidated.

**FIGURE 3:**
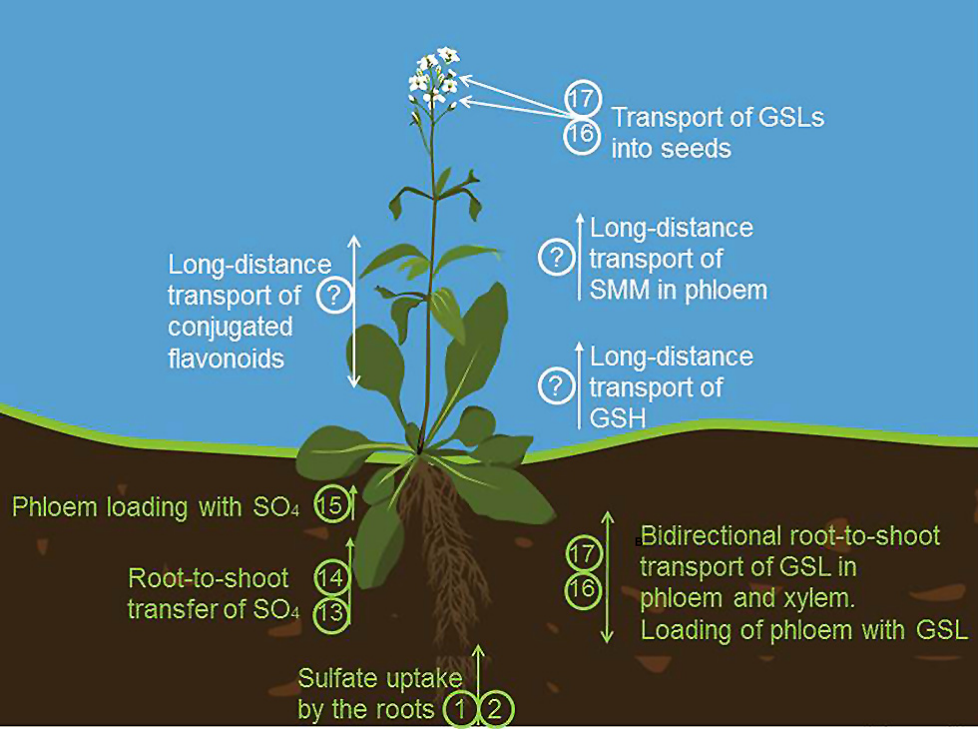
**Long distance transport in sulfur metabolism.** Sulfate is taken up by the roots by SULTR1;1 **(1)** and SULTR1;2 **(2)**. Root-to-shoot transfer of sulfate is facilitated by SULTR2;1 **(13)** and SULTR3;5 **(14)** Transport of sulfate to phloem is possible due to SULTR1;3 **(15)**. **(16)** and **(17)** are transporters of GLS known as GTRs (glucosinolate transporters). They mediate the bidirectional long distance transport of GLS between roots and shoots via both phloem and xylem, facilitate the phloem loading, transport of GSL from the source to the sink tissue and import of GLS into the seeds. *S*-methylmethionine (SMM) and GSH are also important transport form of reduced sulfur found in phloem. How SMM and GSH are loaded into the phloem in plants, remains at the moment unknown. The long distance transporters of flavonoids have been also predicted to exist but still await an identification.

Cys as the first product of sulfate assimilation is used in many metabolic processes. Among the most important sulfur compounds in plant cells derived from Cys is the tripeptide GSH, γ-glutamylcysteinyl glycine (Noctor et al., 2012). GSH is synthesized in two steps from the constituting amino acids, but in this synthesis a transport step is of utmost importance. In contrast to initial reports placing GSH synthesis to both plastids and cytoplasm, the first enzyme of the pathway, γ-glutamylcysteine synthetase, is strictly localized in plastids, at least in *Arabidopsis* (Wachter et al., 2005). GSH synthetase, on the other hand is present in both compartments but mostly in the cytoplasm. The intermediate γ-glutamylcysteine thus has to be exported from the plastids for efficient GSH synthesis (**Figures 1 and 2**, transporter 6). This conclusion has been confirmed by showing that cytosolic expression of GSH synthase rescues the seedling lethal *gsh2* mutant (Pasternak et al., 2008). GSH itself is present in all compartments, with particularly high concentration in the mitochondria (Zechmann et al., 2008). Thus, mitochondrial GSH transporter has to be postulated for plant cells (**Figure 1**, the identity is unknown). In addition, GSH is one of the forms of reduced sulfur subjected to long-distance transport (Herschbach and Rennenberg, 1995), with the need for plasma membrane transporters (**Figures 1 and 3**, the identity is unknown). While the presence of such GSH transporters was long recognized, the molecular nature of such carriers is still far from being determined. The identification of high affinity GSH transporter in yeast (Bourbouloux et al., 2000) triggered the search for plant GSH transporter, particularly as *Arabidopsis* possess nine homologs of the yeast HGT1 (Cagnac et al., 2004). While some transporters of the oligopeptide transporter family indeed transported GSH (Bogs et al., 2003; Cagnac et al., 2004; Zhang et al., 2004), the affinity and specificity was not as high as expected for the high flux of GSH within plant cells. An alternative pathway for GSH transport has been proposed, analogical to the animal gamma-glutamyl cycle, in which GSH is moved across the membrane through combination of degradation, amino acid transport, and synthesis (Meister et al., 1981). The key enzyme of this cycle, the GGT is present in plants and has been shown to be important for recovery of apoplastic GSH (Ferretti et al., 2009), its contribution to total GSH uptake has, however, not been clarified. Since GGT is localized on the apoplast side of plasma membrane or in the tonoplast, it cannot be responsible for the intracellular GSH transport. The first step to understanding the molecular nature of GSH movement between the organelles was identification in *Arabidopsis* of a small family of genes similar to chloroquine-resistance transporter of *Plasmodium falciparum* (Maughan et al., 2010). These CLT were found in a genetic screen using the effect of inhibition of GSH synthesis on root growth (Koprivova et al., 2010; Maughan et al., 2010). All three CLTs are found in chloroplast membranes and affect the distribution of GSH between plastids and cytoplasm (**Figure 1**, transporter 6). They facilitate transport of GSH and γ-EC, probably as export from the plastids (Maughan et al., 2010). Loss of CLTs results in sensitivity to cadmium, increased susceptibility to *Phytophora*, and affects root architecture (Maughan et al., 2010; Schnaubelt et al., 2013). However, *Arabidopsis* mutants devoid of all three CLTs are viable, so alternative transport systems for thiols in the plastid membrane have to exist. Similarly, GSH transporters to the mitochondria await discovery.

Glutathione is not only the most important redox buffer, it is also the precursor for synthesis of PC, small peptides involved in binding and detoxification of heavy metals (Vatamaniuk et al., 1999). Since it had been long known that the last step of detoxification is the transport of the PC-metal complexes into the vacuole, the corresponding transporters have long been sought for (Hall, 2002). In fission yeast, an ABC transporter present in the tonoplast is responsible for such transport and its loss results in cadmium sensitivity (Ortiz et al., 1992). Plant possess a large number of ABC transporters with potentially the same function, therefore, first plant ABC transporters, importing PC-As complexes into the vacuole, have been identified only recently (Song et al., 2010). Loss of two ABC transporters, AtABCC1 and AtABCC2, results in reduced arsenic tolerance, and on the other hand, their expression in yeast increases As tolerance. The AtABCC1 and AtABCC2 are involved also in detoxification of cadmium and mercury (Park et al., 2012). The identification of these transporters is particularly important for engineering heavy metal tolerant and perhaps phytoextracting organisms. PCs are, however, also important for long-distance transport of metals, it remains to be shown, whether the same or other ABC transporter participate in such transport.

## TRANSPORT OF METHIONINE AND ITS DERIVATIVES

Methionine is another S-containing amino acid with a complex demand for inter- and intracellular transfer, particularly when its derivatives are taking into account. Similar to Cys, Met has to be present, and/or transported, to all compartments with protein synthesis (**Figure 1**, the identity is unknown). In addition, traditionally it was thought that Met synthesis in plants requires transport of homocysteine from plastids to the cytoplasm, as the activity of the last enzyme of the biosynthetic pathway, MS, was found in this compartment only (Eichel et al., 1995). However, a plastid localized MS has been described leading to the conclusion that plastids are autonomous for Met synthesis and that cytosolic MS is involved predominantly in regeneration of Met for SAM synthesis in the SAM cycle (Ravanel et al., 2004; Sauter et al., 2013) and for the biosynthesis of Met-derived GSL (Schuster et al., 2006). However, interestingly, no reports on the importance of the plastidial MS and the possibility of complementation of its function by the cytosolic isoform are available. SAM is a methyl donor and cofactor in numerous cellular processes and its synthesis uses some 80% of newly synthesized Met (Hesse et al., 2004). SAM is required in all organelles and has to be transported across plastidic and mitochondrial membranes, since it is synthesized only in the cytoplasm (Wallsgrove et al., 1983; Shen et al., 2002). The SAM transporter (SAMT) has been identified in *Arabidopsis* by Bouvier et al. (2006; **Figure 1**, transporter 7). This transporter, similar to yeast mitochondrial SAM carrier, was shown to be additionally localized in mitochondria, and to contains five transmembrane helices (Bouvier et al., 2006; Palmieri et al., 2006). The SAMT1 catalyzes exchange of SAM with S-adenosylhomocysteine, the by-product of methylation reactions that has to be regenerated to SAM in the cytoplasm. SAMT1 is important, but not essential, as demonstrated by survival and setting seeds, but strong morphological phenotypes of the corresponding T-DNA line (Bouvier et al., 2006). Another Met derivative, SMM is found in the phloem as an important transport form of reduced sulfur (Bourgis et al., 1999; **Figure 3**, the identity is unknown). Plant SMM transporters are not known, but expression of a yeast SMM transporter in pea resulted in improved sulfur and nitrogen content of the seeds (Tan et al., 2010). The yeast SMM transporter is distinct from the SAMT described above and does not have clear homologs in plants (Rouillon et al., 1999). How is SMM unloaded into the phloem in plants, therefore, still needs to be clarified.

## CHLOROPLASTIDIC TRANSPORTER IN THE BIOSYNTHESIS OF MET-DERIVED GSLs

Glucosinolates are a group of amino acid derived secondary compounds, important for plant defense against various pests but also for human nutrition as determinants of taste and smell of crucifers and because of their anticancerogenic properties (Sonderby et al., 2010). Enzymes required for the biosynthesis of GSLs core structure have been identified and shown to be localized in cytoplasmic compartment. In brief, the amino acid is converted to aldoximes by CYP79Fs converting Met derivatives (Hansen et al., 2001; Chen et al., 2003). Next, aldoximes are oxidized to either nitrile oxides or aci-nitro compounds with CYP83A1 (Bak and Feyereisen, 2001; Hemm et al., 2003; Naur et al., 2003). Following fusion of the activated aldoximes to GSH, the produced S-alkylthiohydroximates are converted to thiohydroximates by the C-S lyase SUR1 (Mikkelsen et al., 2004). The last but one reaction in GSL biosynthesis is the S-glucosylation of thiohydroximates by glucosyltransferases of the UGT74 family, with UGT74C1 recently shown to glucosylate Met-derived substrates (Douglas Grubb et al., 2014). The final step of GSL biosynthesis is a SOTs, which form GSL from desulfoGSL. The sulfate for the SOT reaction is provided by PAPS made by APS kinase mainly in chloroplasts (Mugford et al., 2009).

Met is an essential constituent for the production of aliphatic GSLs in *Arabidopsis thaliana*. Before entering the GSL core biosynthetic pathway, Met undergoes chain elongation process in chloroplasts, which is similar to the conversion of the branched-chain amino acid Val to Leu in primary metabolism. This process and its linkage with cytosolic GSL synthesis requires two transport steps across the chloroplast envelope, as the first reaction allowing Met to enter biosynthesis of GSLs is a deamination of Met by a cytosolic BCAT4 (Schuster et al., 2006). Three following reaction are known to take place in chloroplasts, where the respective 2-keto-acid derived from MTOB needs to be imported (**Figure 2**, transporter 8). The MTOB-transporter was identified as BAT5 or BASS5 following its activation *in trans* by R2R3 MYB factors regulating GSL biosynthesis (Gigolashvili et al., 2009). Notably, the reduction of *BAT5* transcripts in *bat5* mutant resulted in up to 50% decreased levels of Met-derived GSLs, indicating a role of BAT5 in the transport of GS intermediates across the plastid envelope (Gigolashvili et al., 2009; Sawada et al., 2009b). Based on the analysis of *bat5,* its metabolic complementation by 2-keto-acids and genetic complementation using chemically inducible promoter fused to *BAT5* CDS, the BAT5 has been proposed as a facilitator of keto-acids across chloroplasts (Gigolashvili et al., 2009; Sawada et al., 2009b). Interestingly, not only MTOB, but also other 2-keto acids like 5-methylthio-2-oxopentanoate (MTOP); 6-methylthio-2-oxohexanoate (MTOH), and 7-methylthio-2-oxoheptanoate seem to be exchanged between plastid and cytoplasm by BAT5. Indeed, feeding of wild-type and *bat5* roots of *Arabidopsis* with 2-keto acids with different chain-length demonstrated that they are substrates of BAT5 since they could be successfully imported into chloroplasts of wild-type plants but not in the *bat5* plants. The proteins homologous to BAT5 in *Arabidopsis* are obviously not important to transport GSL biosynthesis intermediates, as the triple *bat3 bat4 bat5* mutant was not more affected in GSL biosynthesis than the single *bat5* mutant.

Once keto-acids are imported into chloroplasts by BAT5, they enter several cycles of reactions including: condensation with acetyl-CoA catalyzed by a MAM, isomerization by an IPMI, and oxidative decarboxylation by an IPMDH (Gigolashvili et al., 2009; Knill et al., 2009; Sawada et al., 2009a,b). Elongated ketoacid can be again aminated by a BCATs to yield the side-chain elongated Met (homoMet), which is either channeled into GSL biosynthesis or can proceed through another round of chain elongations to yield dihomoMet, trihomoMet, etc. For detailed review see (Grubb and Abel, 2006; Sonderby et al., 2010). The obtained 2-keto-acids with longer carbon chain, or derived amino acid, need to be exported from the chloroplast into the cytoplasm to be incorporated into the GSL core biosynthetic pathway (**Figure 2**, transporter 8 and/or transporter with unknown identity). However, the identity of this transporter, which is involved in the export of amino acid, is unknown. It was also not possible to analyze whether keto-acids or amino acids are preferentially exported from the chloroplasts. Theoretically, the export of both types substrates is possible from chloroplasts as the transamination (and also deamination reactions) of keto acids could be catalyzed by both chloroplastidic BCAT3 (Knill et al., 2008) and cytoplasmic BCAT4 (Schuster et al., 2006). It is rational to suppose that the BAT5, which imports the 2-keto-acid MTOB into the chloroplasts, also exports the chain-elongated 2-keto-acids back into the cytoplasm. However, attempts to measure MTOB and homomethionine transport activity of recombinant BAT5 *in vitro* were not successful till now, as the direct measurements of transport of these substrates in artificial membranes is problematic due to higher hydrophobicity of substrates leading to unspecific membrane binding and permeation, which override the specific transport events (Gigolashvili et al., 2009). New technologies and approaches are needed to find out whether BAT5 is a dual transporter and if not, to identify the exporter of the elongated Met.

## PAPS/PAP ANTIPORTER IN CHLOROPLASTS

Along with the BAT5, which exchanges 2-keto acids between chloroplast and the cytoplasm, the chloroplastidic transporter of activated sulfate (PAPS), which is essential for the sulfation of GSLs, flavonols, peptides, and other sulfated compounds in the cytoplasm has been recently identified (**Figures 1 and 2**, transporter 5). The existence of this transporter has been postulated (Mugford et al., 2009, 2010) based on following observations: (i) There are two parallel plastidic and cytoplasm-localized pathways for the production of PAPS in the cell and both PAPS pathways are capable of providing essential PAPS for the growth and development of plants; (ii) the main pool of PAPS required for the biosynthesis of GSL is produced in chloroplasts, whereas the sulfation of GSLs mediated by SOTs takes place in the cytoplasm; (iii) single mutant missing the cytoplasmic PAPS biosynthetic pathway contain normal levels of GSLs, thus PAPS from the plastid need to be transported into the cytoplasm; (iv) The triple loss-of-function mutants of APK isoforms with sole APK activity localized in chloroplasts was viable due to possibility of PAPS transport/export across chloroplastidic membrane. Finally, the heterologous production of GSLs in *Nicotiana benthamiana* leaves revealed the GSL sulfation as a bottleneck in GSL biosynthesis. This blockage in GSL production could be overcome by the overproduction of PAPS, but not of SOTs, substantiating the importance of activated sulfate and co-regulation of sulfate assimilation with GSL production in the cell (Moldrup et al., 2011).

It therefore has been concluded that there must be a plastidial PAPS transporter to enable PAPS to be delivered to the cytoplasm-localized SOT enzymes. Indeed, it could be recently shown that the transporter formerly known as thylakoid ADP/ATP carrier TAAC (Thuswaldner et al., 2007), has a dual function and transports PAPS across the plastid envelope by favoring PAPS and PAP as substrates. The PAPST1 is a specific transporter of PAPS which acts through an antiport mechanism with PAP as the exchange substrate, thus providing the cytoplasm with activated sulfate and transporting back the toxic by-product of sulfation reaction to chloroplasts. Notably, an enzyme detoxifying PAP into AMP known as FRY1/SAL1 is localized in chloroplasts (Estavillo et al., 2011), and therefore the transport of PAP into chloroplasts is both logical and essential for the cell. Biochemical studies of PAPST protein along with molecular and physiological analyses of *papst1/taac* loss-of-function mutant revealed an impairment of biosynthesis of sulfated compounds and involvement in sulfur metabolism (Gigolashvili et al., 2012). Thus, *papst1/taac* mutant was shown to be slightly retarded in growth compared to corresponding wild-type plants (Col-*0* or WS-*4*; Thuswaldner et al., 2007; Yin et al., 2010) resembling the phenotype of plants lacking the plastidic APK isoforms *apk1apk2* and *apk1apk2apk4* (Mugford et al., 2009, 2010). As for the GSL analysis, *papst1*/*taac* mutant still contained about 30–50% of Met-derived GSLs and no changed levels of indolic GSLs compared to corresponding wild-type plants. As the reduction of GSL levels is not as dramatic as in *apk1apk2* mutants (Mugford et al., 2009) an existence of additional exporter of PAPS in plastid envelopes is expected.

Remarkably, in contrast to PAPS transporters known from mammals, which are members of the nucleotide–sugar transporter family, the chloroplastidic PAPST transporter belongs to the mitochondrial carrier family (Haferkamp et al., 2011).

## PAPS TRANSPORTERS IN GOLGI

Along with chloroplastidic exporter of PAPS, yet unidentified importer of PAPS is expected to exist in Golgi apparatus of plants (**Figure 2**, the identity is unknown). Golgi in plants is known to contain the TPST. TPST catalyzes the post-translational Tyr-sulfation of peptides by the transfer of sulfate from PAPS to the phenolic group of tyrosine (Moore, 2003). In plants, a single copy gene for TPST has been found to have no similarity to cytoplasmic SOTs and no similarities to TPST from yeast or animals (Komori et al., 2009). So far, three families of sulfated growth factors, as substrates of the TPST, have been discovered in plants. These include a disulfated pentapeptide PSK (Matsubayashi et al., 1999, 2006), plant peptide containing sulfated tyrosine 1 (PSY1) containing 18 amino acids (Amano et al., 2007) and root growth factor, also known as CLV3/ESR-related gene family like peptides with 12–13 amino acids (RGF/ CLELs; Matsuzaki et al., 2010). These have been shown to mediate cell proliferation, expansion, and maintenance of the root stem cell niche in *Arabidopsis* (Matsuzaki et al., 2010; Zhou et al., 2010). The *tpst-1* mutant has been shown to exhibit a dwarf phenotype with abnormally root apical meristem, small and pale leaves, and early senescence (Komori et al., 2009). Therefore, like TPST, the Golgi resident PAPS transporter is supposed to have an important function in plant growth and the knockout should phenocopy the *tpst*(s) phenotype. Several Golgi-localized transporters of PAPS have been described in different organisms like *Drosophila* (SLALOM; Luders et al., 2003), human (PAPST1, PAPST2; Kamiyama et al., 2003, 2006), zebrafish (PAPST1; Clement et al., 2008), and *Caenorhabditis* (PST-1; Bhattacharya et al., 2009) but the transporter still awaits identification in plants.

## TRANSPORT OF NATIVE GSLs AND FLAVONOIDS WITHIN THE CELL

Unless sulfated, GSL are not active and cannot be transported within the plant. Thus, sulfation of GSL is a prerequisite for their activity and long distance transport (Mugford et al., 2009). In contrast to GSLs comprising about 200 different structures known at the moment (Clarke, 2010), there are up to 9000 structural variants of flavonoids (Williams and Grayer, 2004) with the modification including *0*-sulfation, *0-*methylation, and *0*-glycosylation. High variety of flavonoids and their modification (Williams and Grayer, 2004) and the absence of universality of flavonoid modifications in different plant species (Varin et al., 1997) makes difficult to predict the role of sulfation. Since the first discovery of flavonoid in 1937, a number of flavonoid sulfates with structural variation were reported in various plant species (Barron et al., 1988). Still, whereas function of sulfated vs. non-sulfated GSLs is well defined at the moment, the information on flavonoid sulfates remains scarce. To better understand the role, activity, and transport of conjugated flavonoids in plants, additional work addressing the sulfation of flavonoids and analysis of loss-of-function mutants of SOTs is needed.

After completion of biosynthesis, secondary metabolites like GSLs and flavonoids, which are present at high concentrations in the cell, need to be imported into the vacuole (**Figure 2**, the identity is unknown), stored there or further metabolized and/or transported (**Figure 3**, transporters 16 and 17). Vacuolar localization of secondary metabolites has been suggested long time ago but it was experimentally demonstrated using water-free environment gradient centrifugation of *Arabidopsis* leaf tissue only recently (Krueger et al., 2011). Shuﬄing of sulfated secondary metabolites into vacuole requires intracellular transporter. Whereas no transporters of native GSL residing in the tonoplast are known at the moment, the vacuolar transporters of flavonoids are known (**Figure 2**, transporters 10 and 11) and we consider them below.

The main driving force for the transport of some flavonoids and anthocyanins into vacuole has been inferred to be a proton gradient between the cytoplasm and the vacuole (or the cell wall), which is maintained by H^+^-ATPases (and H^+^-PPases in the tonoplast; Gomez et al., 2009; Petrussa et al., 2013). Once these compounds are moved into vacuoles, the acidic pH inside the vacuolar compartment and the acylation of flavonoids are both necessary for the induction of a conformational modification, responsible for the appropriate trapping and retention of the metabolites (Kitamura, 2006; Petrussa et al., 2013). Along with a proton gradient force, the ABC transporters have also been reported in sequestration of flavonoids into the vacuole (Yazaki, 2005; Zhao and Dixon, 2010; Petrussa et al., 2013). These proteins are capable of coupling the hydrolysis of ATP to a direct translocation of flavonoids glycosides, glucuronides, GSH conjugates and probably also flavonoid sulfates (Klein et al., 2000; Springob et al., 2003; Rea, 2007; Kang et al., 2011; Martinoia et al., 2012). ABC transporters are ubiquitous ATP-dependent transporters, receptors, and channels that can be found in all three kingdoms of life (Schmitt and Tampé, 2002). The knowledge about plant ABC transporters is rather limited although the genome of *A. thaliana* with 128 ABC genes contains the highest number of genes encoding for ABC transporters compared to other systems, among them 22 members of the MDR, 15 members of the MRP, and 13 members of the PDR family have been annotated (Rea, 2007). For comparison, the human genome contains 48 ABC transporter genes including only two members of the MDR family (Dean and Annilo, 2005). In striking contrast to humans, the majority of MDRs in plants is not located at the plasma membrane but at the vacuolar tonoplast (Rea, 2007). ABC transporters are structurally characterized by two cytosolic nucleotide-binding sites. Their activity is inhibited by vanadate, an inhibitor of P-ATPases and they are insensitive to bafilomycin, a specific inhibitor of V-ATPases (Rea, 2007; Kang et al., 2011).

The involvement of MRP subfamily of ABC transporters (also known as MRP/ABCC and GSH *S*-conjugate pump) in the transport of glutathionylated anthocyanins has been originally reported in maize and petunia (Mueller et al., 2000; Goodman et al., 2004). Interestingly, the mutants defective in GST are unable to accumulate anthocyanins in vacuoles (Marrs et al., 1995; Alfenito et al., 1998; Koes et al., 2005) suggesting that glutathionylation of flavonoids is important for their transport. Based on this observation, the MRP were suggested as major candidates for their translocation of flavonoid conjugates into and out of vacuole. And this seems to be the case also in *Arabidopsis* (Kitamura et al., 2004). Still, GSH conjugation seems to be not always an essential prerequisite for anthocyanin transport mediated by MRP transporters as it could be recently shown that anthocyanidin 3-*O*-glucosides can be transported into microsomes of yeast expressing MDR from grapevine by co-transporting GSH (Francisco et al., 2013).

More on the transport processes in flavonoid biosynthesis has been ensured due to analysis of *Arabidopsis tt* mutants known to be defective in flavonoid biosynthesis (Kitamura, 2006). For example, the mutant *tt12* exhibits pigment deficiency in the seed coat due to the lack of vacuolar deposition of Pas (Debeaujon et al., 2001). The TT12 protein is involved in the uptake of PAs and exhibits sequence similarity to MATE (Debeaujon et al., 2001; Martinoia et al., 2007; Zhao and Dixon, 2009). Remarkably, the transport activity of MATE substrates is dependent on a proton gradient in the opposite direction (Li et al., 2002; Martinoia et al., 2007). Due to more acidic environments of the vacuole compared to the cytoplasm, MATEs are predicted to facilitate both export from plant cells and vacuolar sequestration of their substrates (Yazaki et al., 2008). The genome of *Arabidopsis* contains at least 54 MATE family members but the functional information on these proteins is scarce. MATE family of transporters have been assigned to a larger family of multidrug transporters (Brown et al., 1999). Along with MATEs, the multidrug transporters confer four more superfamilies: the ABC superfamily, the major facilitator superfamily, the small MDR family, and the resistance-nodulation-cell division family (Eckardt, 2001). The MATE family is characterized by the presence of 12 putative transmembrane segments and by the absence of “signature sequences” specific to the other multidrug transporter superfamilies.

Finally, as mentioned before, there is no transporter of GSL into vacuole known at the moment. However, there are some putative transporters of GSL catabolism product belonging to ABC transporter family and PDR subfamily in *A. thaliana* which need to be mentioned here. These candidate transporters have been implied in the transport of Trp-derived GSLs catabolism products and are important for the plant defence (Bednarek et al., 2009; Clay et al., 2009). PDR8/PEN3 and PDR7 have been reported to be localized in plasma membrane of leaves and roots, respectively (**Figure 2**, transporter 9) where they can export yet-unidentified but highly toxic compounds to the site of invasion of non-host pathogens of *Arabidopsis* (Stein et al., 2006; Bednarek et al., 2009). Whereas PDR8/PEN3 has been suggested to be involved in the transport of PEN2 (myrosinase-like protein) catabolism compounds derived from 4-methoxy-indole-3-ylmethyl-glucosinolate (4MOI3M) in leaves, the PDR7 should be transporting products derived from 1-methoxy-indole-3-ylmethyl-glucosinolate (1MOI3M) in roots. Notably, PEN4, which has a similar to PEN2 and PEN3 penetration phenotype associated with the resistance of *Arabidopsis* against non-host pathogen (induced defense of *Arabidopsis* against the grass powdery mildew *Blumeria graminis hordei*) is a phytochelatin synthase, suggesting possible PEN4-mediated glutathionylation of products to be transported by PDR8/PEN3. Another member of the ABC family in wheat known as LR34, controls durable disease resistance against some of the most devastating fungal pathogens such as leaf rust and stripe rust, potentially by exporting other antifungal metabolites although the mechanism is currently not understood (Krattinger et al., 2009).

## TRANSPORT MEDIATED BY VESICLE TRAFFICKING IN PLANT CELLS

Along with the classical transport of metabolites, emerging evidence suggests the participation of a membrane vesicle-mediated trafficking of metabolites (Petrussa et al., 2013). The possible scenario is that different components of the same pathway are localized in different parts of the cell and vesicle movement allows the products to meet when needed. In addition, the end products of biosynthesis could be moved to the storage site as it was shown for flavonoids (Zhang et al., 2006; Poustka et al., 2007; Sun et al., 2012) due to same mechanisms of transport. The vesicles filled with metabolites release their content into the vacuole or apoplast by a fusion with the respective membranes.

Vesicles involved in the transport of flavonoid-derived compounds have been found in maize and in sorghum cells (Snyder and Nicholson, 1990; Grotewold et al., 1998; Grotewold and Davies, 2008; **Figure 2**, transporter 12). It could be further demonstrated that anthocyanins are transported to the cell wall or the vacuole by at least two distinct vesicle trafficking pathways (Lin et al., 2003). The first leads to the accumulation of anthocyanic vacuolar inclusions – the dark red- to purple-pigmented spherical bodies in vacuole known also as AVIs. The second is a TGN-independent pathway, suggesting that it is different from the secretion pathway of most proteins. In accordance with ER-to-vacuole vesicular transport of anthocyanins mediated by a TGN-independent mechanism, Poustka et al. (2007) have demonstrated that the Golgi-disturbing agent has no effect on the accumulation of anthocyanins in investigated cells. Furthermore, it was also showed that cells accumulating high levels of anthocyanins can make use of protein secretory trafficking pathway for the direct transport of anthocyanins from ER to vacuole. More recent works on vesicle trafficking transport mechanisms in *Arabidopsis* reveal that the formation of AVIs is strongly related with the involvement of an autophagic processes (Pourcel et al., 2010; Kulich et al., 2013). The macroautophagy is the best-characterized autophagy pathway in cells. From studies on non-plant organisms we know that the term autophagy includes several mechanisms with the different membrane contributions to build up the phagophore – ER being an important starting compartment for this process (Kulich et al., 2013).

As an example, we can consider above mentioned PEN pathway. Thus, as it was stated in original discovery, the broad-spectrum resistance pre-invasively depends on PEN2 and PEN3 belonging to the same pathway and processing two consecutive steps localized in peroxisome and plasma membrane. It has been therefore postulated that peroxisomes loaded with PEN2 and respective indolic GSL catabolism product move to the site of fungal entry and localization of PEN3/PDR8. This allows the generation of high local concentrations of indolic GSL breakdown products which might then be transferred to the apoplast and side of accumulation of plant pathogens by PEN3 (Lipka et al., 2008; Bednarek et al., 2009).

In addition, a GSH-S-transferase (TT19) in *Arabidopsis* was demonstrated recently to function as a carrier that transports anthocyanin (without glutathionation) from the production site in ER-associated cytoplasm to the tonoplast (Sun et al., 2012). Previously, in *M. truncatula* it was shown that malonylated flavonoid glucosides had increased affinity for MtMATE2 (Zhao et al., 2011) and it was hypothesized that upon malonylation of anthocyanins, the modified anthocyanins were released from TT19 at the tonoplast, and subsequently taken up by tonoplast-localized MATE transporters.

## LONG DISTANT TRANSPORT OF SULFATED GSLs

It has long been known that GSL undergo also a long distance transport, which is needed for loading GSL into the seeds. Radiolabeled GSL applied to leaves of *Brassica napus* were shown to move through the phloem to seeds, where the radioactivity was detected (Brudenell et al., 1999). It was also shown that leaf protoplasts as well as rapeseed embryos incubated in GSL-containing media were able to accumulate GSLs, pointing to a GSL transporter in plasma membrane (Gijzen et al., 1989; Chen and Halkier, 2000). Remarkably, sulfated GSLs and not the desulfoGSLs could be transported into the seeds as revealed by the analysis of APK loss-of-function mutant *apk1 apk2* (Mugford et al., 2009). This mutant possesses strongly reduced levels of sulfated GSLs in leaves and in seeds but desulfoGSL accumulate only in leaves and not in the seeds substantiating the sulfation of GSLs as a prerequisite for their transport.

The long sought transporters of GSLs, facilitating long-distance transport of Met-derived GSLs were identified (Nour-Eldin et al., 2012) and characterized recently (Andersen et al., 2013; **Figure 3**, transporters 16 and 17). Using *Xenopus oocytes* as an expression system it could be shown that GTR1 and GTR2 are high affinity plasma membrane-localized GSL-specific proton symporters (Nour-Eldin et al., 2012). Analysis of loss-of-function mutants of these two transporters revealed that the accumulation of GSL was reduced by 50% in the seeds and was significantly increased in source leaves. The double mutant *gtr1 gtr2* manifested a GSL-free phenotype in seeds and up to 10-fold over-accumulation in source tissue indicating that the transporters are essential for import of GSL into seeds. Investigation of tissue specific expression of GTRs with the thorough analysis of loss-of-function mutants suggested the role of GTR2 in GSL phloem loading, whereas GTR1 is assisting GTR2 in phloem loading but expected to be additionally involved in distributing GSL within the leaf (Nour-Eldin and Halkier, 2013). Finally, as revealed in grafting and feeding experiments *in vivo*, GTR1 and GTR2 are likely to also be involved in bidirectional distribution of GSLs between the roots and rosettes, substantiating the role of both phloem and xylem as their transport pathways (Andersen et al., 2013). GTRs belong to NRT/PTR family.

The NRT/PTRs constitute a large plant transporter family belonging to the ubiquitous POT (Tsay et al., 2007). Plant genomes, encode very high numbers of NRT/PTR transporters when compared with, e.g., humans (in *Arabidopsis* 53 members vs. six in humans). The ability of the GTRs to transport GSL may have arisen through neo-functionalization of NRT/PTR family members. The retained ability of both GTR1 and GTR2 to transport nitrate corroborates such a *de novo* functional specification. Notably, there are more examples of NRT/PTR proteins transporting different types of substrates. Thus, the NRT1.1 which was mainly known as nitrate transporter (Liu et al., 1999) has been recently shown to function as a nitrate-regulated auxin transporter (Krouk et al., 2010). Another example is the NRT1.2, which was previously characterized as a low affinity nitrate transporter (Huang et al., 1999), was recently identified as putative ABA importer (Kanno et al., 2012). From an evolutionary perspective, this shows that members of the NRT/PTR family have evolved to transport nitrate, defense compounds and plant hormones. The mechanism behind these dual substrate specificities is poorly understood (Nour-Eldin and Halkier, 2013). However, in evolutionary context it makes sense, as Met-derived GSLs are a later invention in plant evolution (both in comparison to other types of GSL and other types of secondary metabolites), and during evolution plants made use of already existing transporters from primary metabolism. There are also other existing examples in Met-derived GSL biosynthesis demonstrating the neo-functionalization of genes originally belonging to primary metabolism into secondary metabolism. Thus, the chloroplastidic chain-elongation pathway of Met requires MAM (Field et al., 2006; de Kraker and Gershenzon, 2011), IPMI (Knill et al., 2009), and IPM-DH (Sawada et al., 2009a) and allows in three consequent reactions elongation of Met chain by a single methylene group (–CH2–). These three enzymes seem to be originally derived from primary Leu biosynthesis, and due to neo-functionalization show an activity toward 2-oxo (keto)-acids of Met in GSL biosynthesis. Whereas MAMs seem to be more specific to Met than to Leu, the large subunit of IPMI – the IPMI-LSU1 functions not only in aliphatic GSL biosynthesis but is still active in Leu biosynthesis (Knill et al., 2009; Sawada et al., 2009a). The specific transporter proteins of Trp-derived GSLs are different from characterized GTRs and still await identification.

## LONG DISTANT TRANSPORT OF FLAVONOIDS

Flavonoids are produced at high levels in photosynthetically active organs as the expression of key biosynthetic gene encoding for CHS is dependent on light. These secondary metabolites were also found in heterotrophic root tissue where they contribute to many important functions like lateral development, gravitropic response, legume nodulation, the induction of the hyphal branching of arbuscular mycorrhizal fungi, as well as the response to phosphate starvation and the inhibition of polar auxin transport (Petrussa et al., 2013). Although isoflavonoids has been reported to be produced at some extent in roots (Chen et al., 2011), the vast majority of flavonoids observed in roots should be transported from the green tissue (**Figure 3**, the identity is unknown). A first indication that intercellular or long distance transport may play an important role in the compartmentation of different flavonoids has been obtained in cotyledons and flower buds of *Catharanthus roseus* (Kaltenbach et al., 1999). Furthermore the confocal microscopy analysis of various *Arabidopsis* flavonoid mutants has shown that the flavonoids accumulate inside cells and are not present in regions among cells, pointing to possible symplastic movement of these molecules (Buer et al., 2007). Moreover, using *in vivo* visualization of diphenylboric acid 2-amino ethyl ether (DBPA)-flavonoid conjugates it could be shown that flavonoids can be selectively and unidirectionally transported to specific cell/tissue types and from one organ to another (Buer et al., 2008). It has been therefore suggested that the distribution of flavonoids should be mediated by an active process instead of a passive diffusion, possibly by action of a MRP/ABCC transporter (Petrussa et al., 2013).

## CONCLUSION AND OUTLOOK

Although sulfur-metabolites represent only a small portion of plant metabolites, the pathways of their synthesis are complex and their function and homeostasis require a large number of intra- and intercellular transport steps. Many of these transporters have been characterized, with some of the main gaps having been filled very recently. Thus, the plastidic SULTR has been identified, the GSL transporter found, as well the plastidic PAPS transporter. However, many other transporters still await discovery, above all the transporter responsible for a major flux of sulfate in the cells, the import into vacuole. With better understanding of secondary sulfur metabolism, the need for specific transporters is being recognized, such as the Golgi PAPS transporter, tonoplast GSL, and sulfoflavonoid transporter, and transporters responsible for cellular movement of minor sulfated compounds. Apart from the gene discovery, other questions have to be addressed: the regulation of the transporters of S metabolites, their contribution to the control fluxes of sulfur throughout the plant, both on cellular and whole plant levels, and the role of these transporters in the interactions of plants with other organisms. New tools and approaches, e.g., ^34^S labeling, exploitation of natural variation, and mathematical modeling will be necessary to obtain the comprehensive list of S-metabolite transporters and to understand the role they play in controlling sulfur metabolism and homeostasis in plants. The increased use of these approaches in sulfur research makes hope for a rapid progress in dissection of the network of transporters in sulfur metabolism.

## Conflict of Interest Statement

The authors declare that the research was conducted in the absence of any commercial or financial relationships that could be construed as a potential conflict of interest.

## ABBREVIATIONS

ABC: ATP-binding cassette transporters
APK: adenosine-5′-phosphosulfate kinase
APS: adenosine-5′-phosphosulfate
BAT or BASS: bile acid transporter
BCAT: branched-chain amino acid aminotransferase
CHS: chalcone synthase
CF: conjugated flavonoids
CLT: chloroquine-resistance transporter (CRT)-like transporters
Cys: cysteine
γ-EC: γ-glutamylcystein
GGT: gamma-glutamyl transpeptidase
GSH: glutathione
GSL: glucosinolates
GST: glutathione-S-transferase
GTR: glucosinolate transporter
H_2_S: hydrogen sulfide
IMPI: isopropylmalate isomerase
IPM-DH: isopropylmalate dehydrogenase
Leu: leucine
MAM: methylthioalkylmalate synthase
MATE: multidrug and toxic compound extrusion
Met: methionine
MDR: multidrug resistance family of transporters
MRP: multidrug resistance-associated protein
MTOB: 4-methylthio-2-oxobutanoate
NRT/PTR: nitrate and peptide transport family
OAS: *O*-acetylserine
OASTL: O-acetylserine (thiol)lyase
PAs: proanthocyanidins
PAP: 3′-phosphoadenosine 5′-phosphate
PAPS: 3′-phosphoadenosine-5′-phosphosulfate
PAPST1: 3′-phosphoadenosine-5′-phosphosulfate transporter
PC: phytochelatins
PDR: pleiotropic drug resistance
PSK: disulfated pentapeptide phytosulfokine
PSY1: peptide containing sulfated tyrosine 1
RGFs: root growth factors (peptides containing sulfated tyrosine)
S: sulfur
SAM: S-adenosylmethionine
SAMT: SAM transporter
SMM: S-methylmethionine
SLC13: sodium/sulfate co-transporter
SLC26: sulfate/anion (A^-^) exchanger
SLIM1: transcription factor Sulfate Limitation 1
SOT: sulfotransferase
STAS: domain found in sulfate transporters with a significant similarity to bacterial anti-sigma factor antagonists
SULTR: plant sulfate transporters
SURE: sulfur-responsive element in the promoters of genes
TGN: *trans* Golgi network
TPST: tyrosylprotein sulfotransferase
*tt*: *transparent testa* mutants
Val: valine
YCT1: cysteine transporter
1MO-I3M: 1-methoxy-indole-3-ylmethyl-glucosinolate
4MO-I3M: 4-methoxy-indole-3-ylmethyl-glucosinolate.
